# Bray‐Curtis (AFD) differentiation in molecular ecology: Forecasting, an adjustment (^*A*^ *A*), and comparative performance in selection detection

**DOI:** 10.1002/ece3.9176

**Published:** 2022-09-11

**Authors:** William B. Sherwin

**Affiliations:** ^1^ Evolution and Ecology Research Centre, School of BEES UNSW‐Sydney Sydney New South Wales Australia

**Keywords:** adaptation, allele frequency difference, biodiversity, genetic differentiation, mutual information, outlier loci

## Abstract

Geographic genetic differentiation measures are used for purposes such as assessing genetic diversity and connectivity, and searching for signals of selection. Confirmation by unrelated measures can minimize false positives. A popular differentiation measure, Bray‐Curtis, has been used increasingly in molecular ecology, renamed AFD (hereafter called *BCAFD*). Critically, *BCAFD* is expected to be partially independent of the commonly used Hill “Q‐profile” measures. *BCAFD* needs scrutiny for potential biases, by examining limits on its value, and comparing simulations against expectations. *BCAFD* has two dependencies on within‐population (alpha) variation, undesirable for a between‐population (beta) measure. The first dependency is derived from similarity to $G_{\mathrm{ST}}$ and $F_{\mathrm{ST}}$. The second dependency is that *BCAFD* cannot be larger than the highest allele proportion in either location (alpha variation), which can be overcome by data‐filtering or by a modified statistic ^*A*^ *A* or “Adjusted AFD”. The first dependency does not forestall applications such as assessing connectivity or selection, if we know the measure's null behavior under selective neutrality with specified conditions—which is shown in this article for ^*A*^ *A*, for equilibrium, and nonequilibrium, for the commonly used data type of single‐nucleotide‐polymorphisms (SNPs) in two locations. Thus, ^*A*^ *A* can be used in tandem with mathematically contrasting differentiation measures, with the aim of reducing false inferences. For detecting adaptive loci, the relative performance of ^*A*^ *A* and other measures was evaluated, showing that it is best to use two mathematically different measures simultaneously, and that ^*A*^ *A* is in one of the best such pairwise criteria. For any application, using ^*A*^ *A*, rather than *BCAFD,* avoids the counterintuitive limitation by maximum allele proportion within localities.

## Graphical abstract

The popular Bray‐Curtis measure has recently been introduced into molecular ecology, including for assessments of connectivity, and to search for genes under selection, both important for evolution and conservation. Unlike similar measures, Bray‐Curtis did not have a theoretical basis, until provided by this article, along with a bias‐correction that allows comparative use, and improves its performance at identifying genes under selection. The figure shows simulations of the corrected measure, with exact fit to the neutral theoretical predictions.

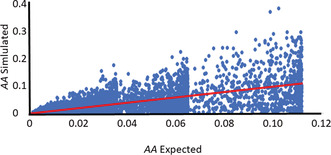

## 1 INTRODUCTION

Comparisons of biodiversity between regions are important aspects of understanding both ecological and genetic systems. There are many geographic differentiation measures, used for purposes such as assessing genetic diversity and connectivity (Gruber et al., 2018; Guillot et al., 2005; Manni et al., 2004; Meirmans, 2020; Sherwin et al., 2017, 2021) and searching for signals of different selective regimes geographically, which is expected to have high false‐positive rates (Bierne et al., 2013; Lotterhos & Whitlock, 2014; Narum & Hess, 2011; Schneider et al., 2021; Xiang‐Yu et al., 2016). Because of the anticipated high false‐positive rates, it is important to confirm findings using a wide range of mathematically unrelated measures. Often these measures are chosen from the Hill “Q‐profile,” which includes: counts or sharing of allelic types (Q = 0 measures); Shannon information and differentiation (Q = 1); and heterozygosity, nucleotide diversity, Gini‐Simpson, $G_{\mathrm{ST}}$, $F_{\mathrm{ST}}$, Morisita‐Horn, $D_{\mathrm{EST}}$ (Q = 2) (Chao et al., 2014; Gaggiotti et al., 2018; Jost, 2008; Jost et al., 2010; Sherwin et al., 2017, 2021). However, despite their different sensitivity to some matters, such as rare and common alleles, the members of the Q‐profile are all mathematically related (Sherwin et al., 2017, 2021).

Notably, one recent addition to the range of measures in molecular ecology is outside the Hill Q‐profile: the Bray–Curtis index of dissimilarity, a method of assessing differentiation that is extremely popular in its original use, to assess differentiation between species assemblages (Bray & Curtis, 1957). During 2021 alone, Bray‐Curtis was mentioned over 10,000 times in Google Scholar. Bray‐Curtis can be expressed in a way that facilitates comparison with differentiation measures derived from Hill numbers; the mathematical equivalence to other formulations of Bray‐Curtis is documented in (Chao & Chiu, 2016; Jost et al., 2010; Ricotta et al., 2021; Ricotta & Pavoine, 2022; Ricotta & Podani, 2017).

(1) $$\text{Bray}-\text{Curtis}=\frac{\sum_{j=1}^{S}\left|a_{1j}-a_{2j}\right|}{\sum_{j=1}^{S}\left(a_{1j}+a_{2j}\right)}$$

where $a_{1j}$ and $a_{2j}$ are the abundances (counts or frequencies 0≤a≤∞) in each of two locations (1,2), for variant *j* (1≤j≤S) and *S* is the total number of species. This measure satisfies many of the requirements of a good measurement of differentiation between assemblages (Chao & Chiu, 2016; Magurran, 2004; Ricotta & Podani, 2017). This index is also used for analysis of operational taxonomic units in metagenomics (Peng et al., 2020).

Unification of ecological and genetic approaches is desirable, because of their interaction as parts of the same biological systems, and because of their underlying mathematical similarities (Rosindell et al., 2015; Sherwin, 2018), so it is good to see that a simplified version of Bray‐Curtis has been proposed as a measure of differentiation in molecular ecology and evolution (Berner, 2019a, 2019b; Shriver et al., 1997), echoing a similar measure in community ecology (Whittaker, 1975) (p 118). It was renamed “allele frequency difference” AFD, but I will call it *BCAFD*, in deference to its original proponents, and because it is a difference of proportions (0≤p≤1) rather than frequencies (0≤a≤∞). In the two‐variant two‐location case, Bray‐Curtis simplifies to the unsigned difference between locations 1 and 2 of proportions of either of the two allelic variants (Berner, 2019a, 2019b).

(2 and A1.1) $$\text{BCAFD}=|p_{1}-p_{2}|$$

where $p_{1}=a_{1}/\left(a_{1}+a_{2}\right)$ and $q_{1}=1-p_{1}$, and similarly for the other location $p_{2}$ and $q_{2}$. When there are multiple alleles, it is suggested to use the sum of the absolute allele proportion differences divided by two ([Berner, 2019a], Table S1), which actually is equivalent to the more general Equation (1). However, unless otherwise stated this article will deal with the biallelic case which is very common in current molecular ecology—SNPs or single‐nucleotide polymorphisms.

In the molecular ecology literature, *BCAFD* has been used or mentioned many times since Berner's publications (Berner, 2019a, 2019b), including for assessment of population differentiation in time or space, with implications for likely genetic connectivity (Amos, 2021; Lou et al., 2021; Popovic et al., 2021; Subramanian, 2021; Taylor et al., 2021; Weldekidan et al., 2022; Wolf et al., 2021), as well as identifying candidate adaptive loci by their strong differentiation relative to other presumably neutral loci (Bharti et al., 2021; Boyle et al., 2021; Haenel, Guerard, et al., 2021; Haenel, Oke, et al., 2021; Price et al., 2020; Yin et al., 2021; Zhou et al., 2021).

For applications including selection detection and assessment of connectivity between locations, it is critical to know the measure's null behavior, that is, in the absence of selection (“neutrality”), with specified conditions such as population size, dispersal, and mutation (Bierne et al., 2013; Gruber et al., 2018; Guillot et al., 2005; Lotterhos & Whitlock, 2014; Manni et al., 2004; Meirmans, 2020; Narum & Hess, 2011; Schneider et al., 2021; Sherwin et al., 2017, 2021; Xiang‐Yu et al., 2016). Despite not belonging to the Hill Q‐profile, *BCAFD* appears to have some mathematical relationship to two of the Hill measures: $G_{\mathrm{ST}}$ and $F_{\mathrm{ST}}$ (Appendix 1). Therefore, based on forecasts for those two measures, it will be shown that it is possible to develop forecasts for *BCAFD* for two‐location, two‐variant systems such as single‐nucleotide polymorphisms (SNPs).

All diversity measures must be scrutinized for their particular properties (Leinster & Cobbold, 2012; Leinster, 2021; Sherwin et al., 2017; Sherwin et al., 2021). An important property of differentiation measures is independence between alpha (within location) variation, beta (between location) differentiation, and total (gamma) variation (Chao et al., 2014; Gaggiotti et al., 2018; Jost, 2008; Jost et al., 2010; Leinster, 2021; Sherwin et al., 2017; Sherwin et al., 2021). Critically, $G_{\mathrm{ST}}$ and $F_{\mathrm{ST}}$ are well‐known to have the serious limitation of being heavily influenced by within‐location variation (alpha), something that is not desirable in a between‐location (beta) differentiation measure. Although $F_{\mathrm{ST}}$ was explicitly proposed as a measure of between‐subgroup differentiation (Wright, 1943) and has been used for that extensively, unlike some other Hill‐profile measures, $F_{\mathrm{ST}}$ shows strong dependence on alpha within‐locality diversity, as does the related measure $G_{\mathrm{ST}}$ (Jost, 2008; Meirmans & Hedrick, 2010; Nei, 1977, 1973). Because of its relationship to $G_{\mathrm{ST}}$ and $F_{\mathrm{ST}}$, it is likely that there will be dependency of *BCAFD* on alpha variation.

Another dependency of *BCAFD* on alpha variation is that it is obvious from Equation (2) that *BCAFD* can never be larger than $p_{\max}$, the higher of the two allele proportions, $p_{1}$ and $p_{2}$. In other words, if either $p_{1}$ or $p_{2}$ is zero, then the value of Bray‐Curtis will be equal to the other, more abundant, proportion. Of course, the values $p_{1}$ and $p_{2}$ are within‐location proportions of one of the two alleles—a within‐population (alpha) measure. This is an extremely counterintuitive limitation on a between‐location (beta) differentiation measure, and is expected to result in biased values. This might be particularly important when using the measure to search for loci that experience different directions of selection in different locations, because this difference of selective regime will obviously give a signal of large differentiation values between locations, relative to other neutral loci. As a result, the truncation of large values of BCAFD due to $p_{\max}$ might be expected to reduce the ability to distinguish such adaptive loci from neutral loci.

The confounds with alpha variation due to relationship to $G_{\mathrm{ST}}$, and restriction by maximal allele proportion $p_{\max}$, require examination in this article; however another possible confound does not appear to be of concern. As well as the proportions of variants, a between location (beta) differentiation measure can be confounded by the number of variant types. This confound can be avoided by restriction to two‐variant systems such as SNPs, as is done in this article. Also, it does not appear to be a problem for the multiallelic version of *BCAFD* (Equation (1), also [Berner, 2019a] Table S1). When there is maximal differentiation, that is, no alleles shared between locations, one expects to always get the maximal value for the genetic differentiation statistic. This in fact does happen. For example, if there are four alleles *w, x, y, and z*, with *w* and *x* in location 1, and the other two in location 2, so that $p_{1w}=p_{1x}=p_{2y}=p_{2z}=0.5$, and other proportions are equal to zero, then the multiallelic statistic is equal to *BCAFD* = 1.0. Also, if location 1 only has allele *w*, and the other three alleles are in location 2, with $p_{1w}=1;p_{2x}=p_{2y}=p_{2z}=\frac{1}{3}$, then the multiallelic statistic remains *BCAFD* = 1.0, as expected for the same situation of maximal differentiation (no shared alleles).

Irrespective of these confounds, it should be noted that the alpha‐dependency of $G_{\mathrm{ST}}/F_{\mathrm{ST}}$ does not forestall all use of these measures, provided that we know their behavior under selective neutrality with specified conditions such as population size, dispersal, and mutation (Bierne et al., 2013; Gruber et al., 2018; Guillot et al., 2005; Lotterhos & Whitlock, 2014; Manni et al., 2004; Meirmans, 2020; Narum & Hess, 2011; Schneider et al., 2021; Sherwin et al., 2017, 2021; Xiang‐Yu et al., 2016). With this in mind, and responding to the increased use of *BCAFD* in molecular ecology described above, this paper carries out the following tasks:

- It creates a modified version of *BCAFD* termed ^*A*^ *A* (“Adjusted AFD”) that is corrected for the limitation by $p_{\max}$.
- Forecasts are made and tested, for Bray‐Curtis (*BCAFD*) and ^*A*^ *A*, for selectively neutral biallelic SNPs—a very common data type at present—under various scenarios of population size, mutation, and dispersal. This will allow *BCAFD*, and especially ^*A*^ *A*, to be used for evaluating competing models of population connectivity, making projections for the future, or identifying outlier loci whose differentiation level departs from neutral expectations, and so are candidate adaptive loci.
- Simulations are performed to investigate how the ^*A*^ *A* correction for bias performs in detecting loci under directional selection, in comparison to competing measures, or in consort with those measures.

## 2 MATERIALS AND METHODS

Forecasting equations for Bray‐Curtis were developed for the common and simple case of a single neutral biallelic SNP locus, with two locations (1,2); the measure can be averaged over multiple loci, and can be applied to haploids, or to diploids in Hardy–Weinberg equilibrium (i.e., no population‐wide correlation between the two alleles within diploid genotypes). When there are only two variants, the Bray‐Curtis equation is: $\text{BCAFD}=\left|p_{1}-p_{2}\right|$ (Berner, 2019a, 2019b) (Equation 2, above) where *p*_*1*_ and *p*_*2*_ are proportions of one of the two alleles at each location ($q_{1}=1-p_{1};q_{2}=1-p_{2}$).

The quantity in Equation (2) is a transform of two well‐known differentiation measures (Halliburton, 2004; Wright, 1943):

(3 and A1.2) $$G_{\mathrm{ST}}=\left[H_{T}-\bar{H_{1},H_{2}}\right]/H_{T}\approx F_{\mathrm{ST}}=\sigma_{p}^{2}/\bar{p}\;\bar{q}$$

where $\sigma_{p}^{2}$ is the variance of *p* between locations, *H* is the Hardy–Weinberg (Binomial) expected heterozygosity, for example, $H_{T}=1-{\bar{p}}^{2}-{\bar{q}}^{2};H_{1}=1-p_{1}^{2}-q_{1}^{2}$; and $\bar{p}$ is the average *p* over the two locations (1,2); $\bar{q}=1-\bar{p}$. The measures $G_{\mathrm{ST}}$ and $F_{\mathrm{ST}}$ in Equation (3) are identical in the two‐allele, two location case ([Halliburton, 2004] Box 9.5). Appendix 1 shows that

(4 and A1.4) $${\text{BCAFD}}^{2}=4\bar{p}\;\bar{q}G_{\mathrm{ST}}=2H_{T}G_{\mathrm{ST}}$$

Because of its close relationship to *G*_*ST*_ or *F*_*ST*_, *BCAFD* forecasts can be based on well‐known forecasts for those measures (Appendix 1). The expectation for diploid *BCAFD* at drift‐dispersal‐mutation equilibrium is:

(5 and A1.7) $$\text{BCAFD}=\sqrt{\frac{2{\;}^{2}D-2}{{\;}^{2}D\left(1+8N\left(2m+\mu \right)\right)}}$$

where *m* is symmetrical dispersal between the two locations (0 ≤ *m* ≤ 1); *μ* is the rate of mutation (0 ≤ *μ* ≤ 1); *N* is the effective population size at each location (identical); and ${\;}^{2}D$ is the second order Hill diversity, or effective number of alleles ${\;}^{2}D=1/\left(1-H_{T}\right)$.

The equivalent equation for the haploid SNPs simulated in this article is:

(6 and A1.8) $$\text{BCAFD}=\sqrt{\frac{2{\;}^{2}D-2}{{\;}^{2}D\;\left(1+4N\left(2m+\mu \right)\right)}}$$

The performance of these equations was assessed by simulation of biallelic neutral single‐nucleotide polymorphisms (SNPs) in two haploid subpopulations, for a wide range of scenarios covering all possible combinations of three symmetric dispersal rates (*m* = 0.01, 0.03, 0.1) and three subpopulation effective sizes (*N* = 1000, 10,000, 100,000). Starting allele proportions in each subpopulation (*p* values) were randomized in each replicate. Simulations used the typical SNP mutation rate (*μ* = 10^−9^), but essentially identical results were obtained with rates between *μ* = 10^−6^ and 10^−12^. The simulation was programmed in MATLAB, and full details are in Appendix 2, and Dewar et al. (2011). There were 1000 replicate iterations of each scenario, which could also be considered as 1000 independently inherited loci (i.e., in linkage equilibrium). Each iteration was run for 200 generations, and each generation included stochastic binomial sampling of the parents' alleles to establish the allele proportions for the offspring, followed by symmetrical dispersal to create the parent populations for the next generation. Because the forecasts are for drift‐dispersal‐mutation equilibrium, it is important to know whether the simulations had reached equilibrium. The adequacy of the run‐time of 200 generations was confirmed in three ways, detailed in Appendix 2: 200 generations was several times longer than the expected time to half‐equilibrium values; inspection ensured an asymptote to a stable value for *BCAFD*; and the variance of *BCAFD* between‐generations was much lower than variance between replicate iterations (typically one tenth or less). The performance of the simulation was checked by comparison with results of EASYPOP (Balloux, 2001) and with known predictions for $G_{\mathrm{ST}}$ (see Appendix 2 for details).

To assess whether the expectation from Equation (6) was an adequate forecast of *BCAFD*, *BCAFD* was calculated at the final generation, then linear regression was used (in EXCEL). If the expectation from Equation (6) is accurate, it is expected that a regression of the simulated *BCAFD* against the expected *BCAFD* should have a slope of unity and an intercept of zero. Additionally, alpha‐dependence was assessed, and possible corrections suggested, including an adjusted measure ^*A*^ *A* that has no limitation by $p_{\max}$.

In other investigations, I examined the relationship between *BCAFD* and three other differentiation measures: $G_{\mathrm{ST}}$, $D_{\mathrm{EST}}$, and mutual information, *I* (Sherwin et al., 2017, 2021). I also examined whether the forecasts could be made completely independent of within‐location variation. Finally, I produced nonequilibrium forecasts, suitable for situations where there has been recent disturbance to connectivity, for example.

Simulations were used to investigate the effect of the adjusted measure ^*A*^ *A* on detectability of loci under different directional selection in each population. These simulations were identical to the ones described above, with two alterations. First, the simulations were restricted to large population size and low dispersal (*N* = 100,000, *m* = 0.01). Second, selection was simulated each generation by, in one location, increasing the number of surviving progeny of one genotype by multiplying by a factor of 1 + *s*/2, and decreasing the same genotype by 1 − *s*/2 in the other location (*s* = 0.001, 0.003, 0.005, 0.05). The highest selection strength (*s* = 0.05) would be expected to result in very high differentiation after the 200 generation simulation period. At the final generation, the program calculated the genetic differentiation measures: ^*A*^ *A*; BCAFD; $G_{\mathrm{ST}}$; $D_{\mathrm{EST}}$ (Jost, 2008); and mutual information I (Sherwin et al., 2017, 2021). For each measure, I tallied the percentage of loci (out of 1000 simulated) that would be identified as outliers (i.e., potentially under selection) using the “univariate” criterion that their genetic differentiation values were in the top 1% of the 1000 loci simulated without selection in a parallel neutral simulation, separately for each one of the five differentiation measures. As well as those univariate criteria, the same analysis was repeated using a series of more restrictive “bivariate” criteria, that is, that for a locus in the selection simulation to be identified as an outlier, it was required to have differentiation in the top 1% of neutral loci for each of a pair of the differentiation measures listed above. For each of these diagnoses (univariate or bivariate), the true positive (TP) was the number of loci known to be under selection that were actually identified as being under selection, out of the total of 1000 independent loci simulated with selection. The false positive (FP) was the number of loci identified as being under selection in the parallel neutral simulation, again of 1000 loci; with the univariate criteria this of course must be 1%of 1000 = 10 loci, but the bivariate criteria are expected to be more restrictive, giving lower FP. Then I calculated a performance value separately for each strength of selection. The performance value is the percentage of loci that are true positive, out of all loci that were identified as outliers potentially under selection (TP + FP); in the case that 1% of all loci were under that selective regime, and all other loci were neutral, the calculation is 100 × (TP × 0.01)/[(TP × 0.01) + (FP × 0.99)]. Of course, the proportions of neutral and selected loci would not be known beforehand, but given that the analysis is standardized to a constant univariate FD rate, the performance values can be used to compare the performance of the different criteria.

## 3 RESULTS

**FIGURE 1:**
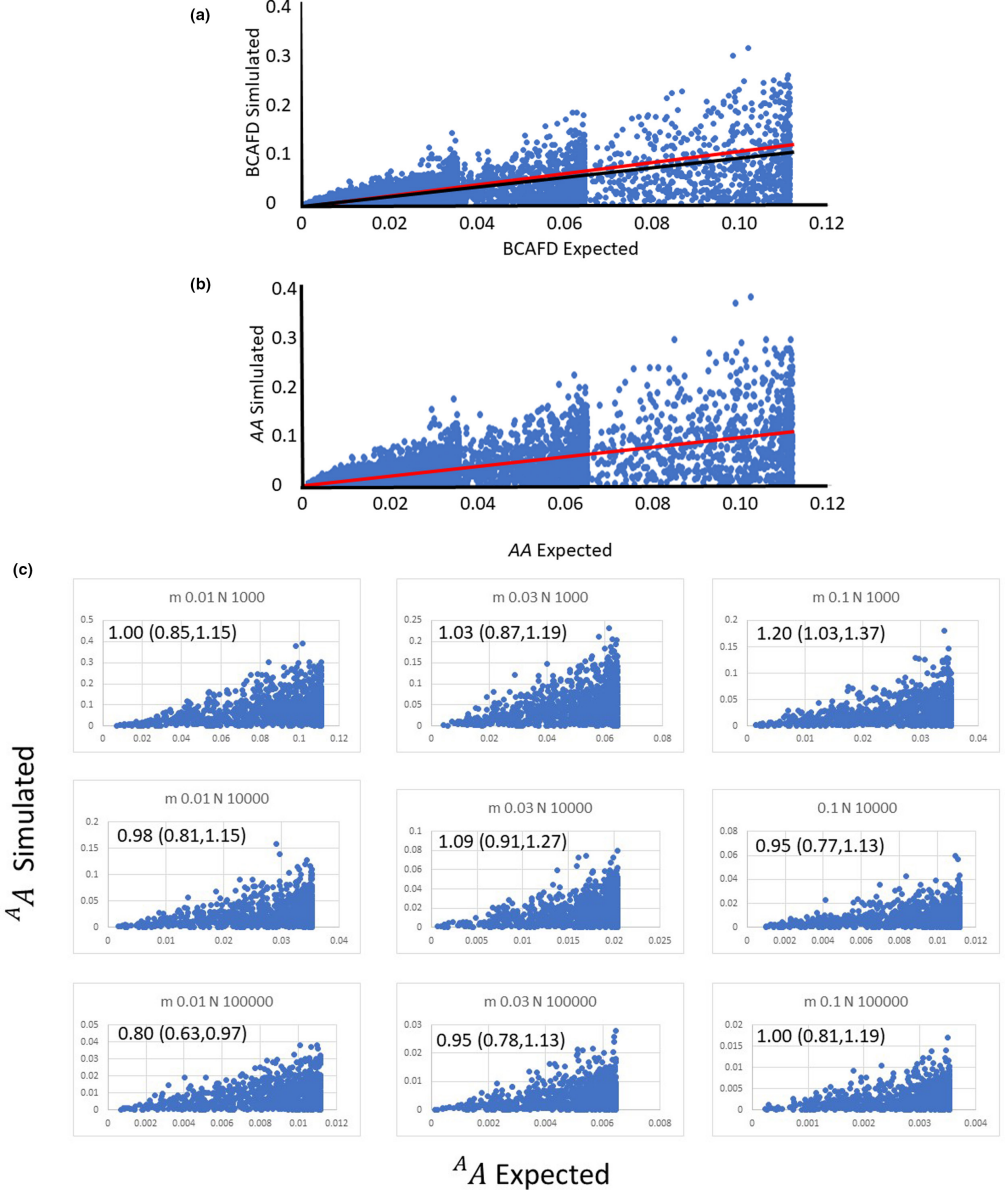
(a) Comparison of simulation results with algebraic predictions for *BCAFD*; 9000 points from the 1000 replicates of each of nine neutral scenarios (effective size *N* = 1000, 10,000, 100,000, dispersal rate *m* = 0.01, 0.03, 0.10) and with regression equation (Simulated‐*BCAFD*) = 0.83 × (Predicted‐*BCAFD* from Equation 6) (significance *P* <<0, *R* ^2^ = .50, intercept negligibly different from zero: −7.6 × 10^−5^). The black line is the regression line; the red line is the expected 1:1 relationship. (b) The same data again, using the correction for the limitation by maximum *p*, that is a plot of ^*A*^ *A* = |*p*_1_ –*p*_2_|/(0.6152 + 0.3985 × *p*_max_) against the expectation shown in Equations (6) and (8). In this case, the expected 45‐degree plot is achieved exactly, with the expected slope of unity (slope coefficient = 1.00, 95% confidence limits 0.98 to 1.02, significance *P* <<0, *R* ^*2*^ = 0.50, intercept negligibly different from zero: 0.0004). The red line for 1:1 slope is exactly coincident with the regression line. (c) The nine scenarios from (b) plotted individually—comparison of simulation results with algebraic predictions, using ^*A*^ *A*, the correction for the limitation by maximum *p*. Each panel shows 1000 points from the 1000 replicates of one scenario, whose dispersal rate *m* and effective size *N* is shown in the panel's headline. The slopes of regression lines are shown on the panels, with 95% confidence intervals, which included unity in all except two marginal cases, and are therefore each concordant with the overall result shown in (b) and the relationship in Equation (8). In all cases, the intercept was negligibly different from zero, and *P* for significance was <10^−18^.

Trials of Equation (6) used the data from the haploid simulation program described above. Figure 1a shows simulated *BCAFD* (Equation 2), calculated for all 9000 datapoints (nine scenarios × 1000 replicates) regressed against algebraic predictions (Equation 6) of *BCAFD* for each replicate in each scenario (again 9000 points). The predictions have to be made separately for each replicate because the stochastic nature of the simulations results in each replicate having a different final value for ${\;}^{2}D$, which is used in Equation 6. Five things are apparent in Figure 1a:

- there are distinct clumps of points, which identify limits when $\bar{p}=\bar{q}=0.5$ so that ${\;}^{2}D=2$, which gives maximum expected *BCAFD* values of 0.035 when $N_{e}m\geq 100$, 0.064 when $N_{e}m=30$, and 0.111 when $N_{e}m=10$ (Equation 6).
- there appears to be an oblique upper bound to the scatter of points from the 1000 replicates of each scenario; this will be discussed later.
- Despite the scatter of replicates, there is an extremely good regression of simulated *BCAFD* on predicted *BCAFD* (significance *P* was extremely low—assigned to zero by the program, see caption of Figure 1a). Note that the scatter is not unexpected given that the initial allele proportions were randomized.
- the intercept is extremely close to zero, as expected
- however, the slope is slightly below the expected 45‐degree line for perfect prediction, with a slope of 0.83, see caption of Figure 1a; the 95% confidence limits for the slope were 0.81 to 0.85, so that the limits did not include the expected unity.

**FIGURE 2:**
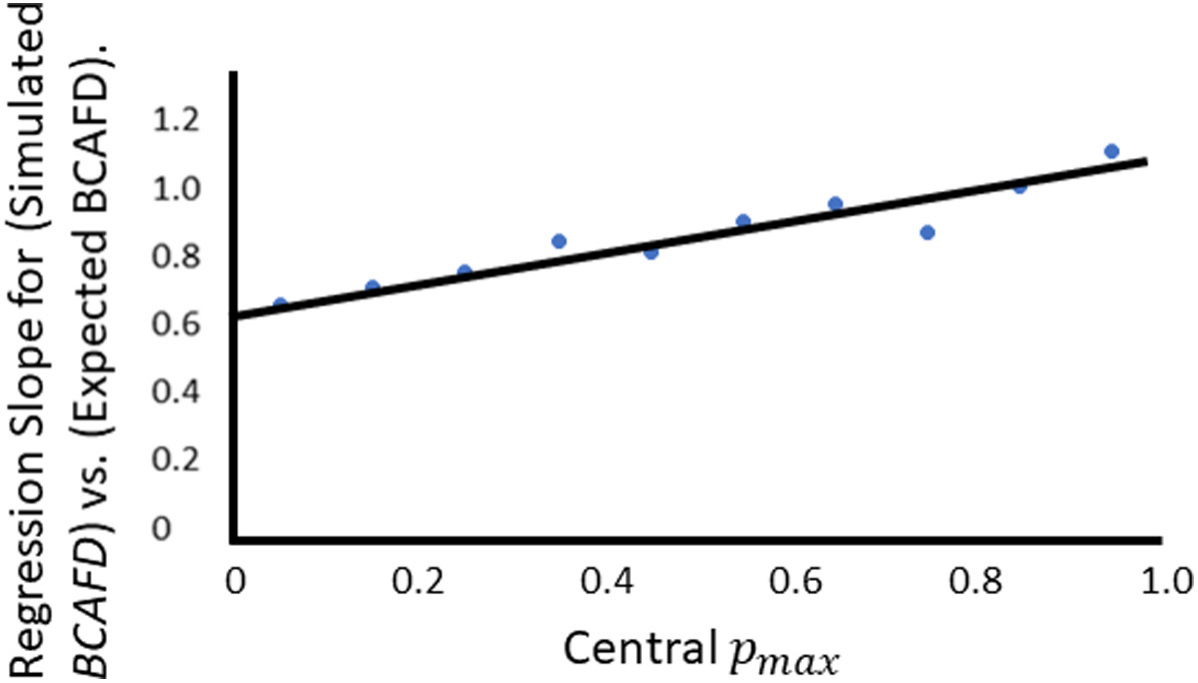
The effect of maximum *p*‐value $p_{\max}$ on the regression slope coefficient of (simulated *BCAFD*) on (expected *BCAFD* from Equation 6). This plot itself has a regression equation: $\left(\text{coefficient of simulated}\;\text{BCAFD}\;\mathrm{on}\;\text{expected}\;\text{BCAFD}\right)=0.6152+0.3985\times p_{\max}$, with $R^{2}$ = .90, and *P* = .000025. The values upon which the plot is based are taken from Table 1.

In the introduction it was pointed out that the value of *BCAFD* is restricted by the maximum *p* value $p_{\max}$ in either of the two locations, at the generation where *BCAFD* is calculated. This is a potential reason for the oblique upper bound for the observations in Figure 1a. To Investigate this, the regression of simulated *BCAFD* on expected *BCAFD* was repeated on ten subsets of the 9000 datapoints, subdivided by the final value of $p_{\max}$, the maximum *p* in either of the two locations. Results in Table 1 show that the departure from a 1:1 slope is indeed due to the restriction by $p_{\max}$. The bottom two rows of this table are where there is the least constraint on simulated *BCAFD* values ($0.8\leq p_{\max}\leq 0.899$ and $0.9\leq p_{\max}\leq 1$), and in these two cases the slope of the regression of simulated *BCAFD* on expected *BCAFD* is indeed unity as expected. The slope of this regression decreases linearly when it is more constrained, that is, with lower $p_{\max}$ values (Table 1 and Figure 2).

**TABLE 1:** The effect of $p_{\max}$ on forecasts for *BCAFD*

Central $p_{\max}$	$R^{2}$	*P* for significance	Intercept	Slope coefficient (95% CL)
0.05	.465	1.0 × 10^−131^	+0.0008	0.630258 (0.59–0.67)
0.15	.444	1.7 × 10^−111^	+0.0020	0.673769 (0.62–0.72)
0.25	.420	2.3 × 10^−104^	+0.0022	0.713834 (0.66–0.77)
0.35	.456	8.2 × 10^−118^	+0.0007	0.79615 (0.74–0.85)
0.45	.414	2.2 × 10^−106^	+0.0024	0.766259 (0.71–0.83)
0.55	.482	4.5 × 10^−126^	+0.0001	0.849727 (0.79–0.91)
0.65	.569	7.4 × 10^−158^	−0.0015	0.900086 (0.85–0.95)
0.75	.482	2.8 × 10^−128^	−0.0008	0.824037 (0.77–0.88)
0.85	.538	1.6 × 10^−151^	−0.0020	0.947642 (0.89–1.01)
0.95	.586	2.1 × 10^−201^	−0.0023	1.042645 (0.99–1.10)

*Note*: The 9000 data points from Figure 1a, sorted by $p_{\max}$ in the final generation. In the first column, “Central $p_{\max}=0.05$” identifies the points with $0\leq p_{\max}\leq 0.099$, etc. The remaining columns show the results of regression analysis of (Simulated‐*BCAFD*) against (Predicted‐*BCAFD* from Equation 6) for the subset of the datapoints identified in the left column. All regressions showed an intercept very close to zero, as expected. Large numbers of significant digits are retained in the slope coefficients because of their subsequent use in the analysis in Figure 2, where the coefficients are plotted against central $p_{\max}$ values.

There are two possible corrections for this dependency on maximum *p* value. First, the data could be filtered to only include loci with very high maximum *p* values ($0.8\leq p_{\max}\leq 1$, Table 1, Figure 2), but of course this would greatly reduce the usable data. Second, because the regression in Figure 2 is very linear, one can correct the expectations for the effect seen in that figure, where

*(coefficient of simulated BCAFD on expected BCAFD)*  = 0.6152 + $0.3985\times p_{\max}$, so that we create a modified version of *BCAFD*, called “^*A*^ *A*” which is free of dependence upon $p_{\max}$ :

(7) $${\;}^{A}A=\frac{\text{BCAFD}}{\left(0.6152+0.3985\times p_{\max}\right)}=\frac{\left|p_{1}-p_{2}\right|}{\left(0.6152+0.3985\times p_{\max}\right)}$$

We then find that the forecasts are general for all values of $p_{\max}$, for haploid:

(8) $$\begin{array}{c} {\;}^{A}A=\frac{\left|p_{1}-p_{2}\right|}{\left(0.6152+0.3985\times p_{\max}\right)}=\sqrt{\frac{2{\;}^{2}D-2}{{\;}^{2}D\left(1+4N\left(2m+\mu \right)\right)}} \end{array}$$

or the same for diploid loci in Hardy–Weinberg equilibrium, replacing 4N with 8N:

(9) $$\begin{array}{c} {\;}^{A}A=\frac{\left|p_{1}-p_{2}\right|}{\left(0.6152+0.3985\times p_{\max}\right)}=\sqrt{\frac{2{\;}^{2}D-2}{{\;}^{2}D\left(1+8N\left(2m+\mu \right)\right)}} \end{array}$$

Figure 1b shows the plot of ^*A*^ *A* (i.e., *BCAFD* adjusted to compensate for limitation by $p_{\max}$) plotted against the expectations from (Equation 8). This regression shows the expected slope of unity and intercept of zero, demonstrating that the simulation confirms the haploid prediction for ^*A*^ *A* in Equation (8), including for each individual scenario (Figure 1c).

**FIGURE 3:**
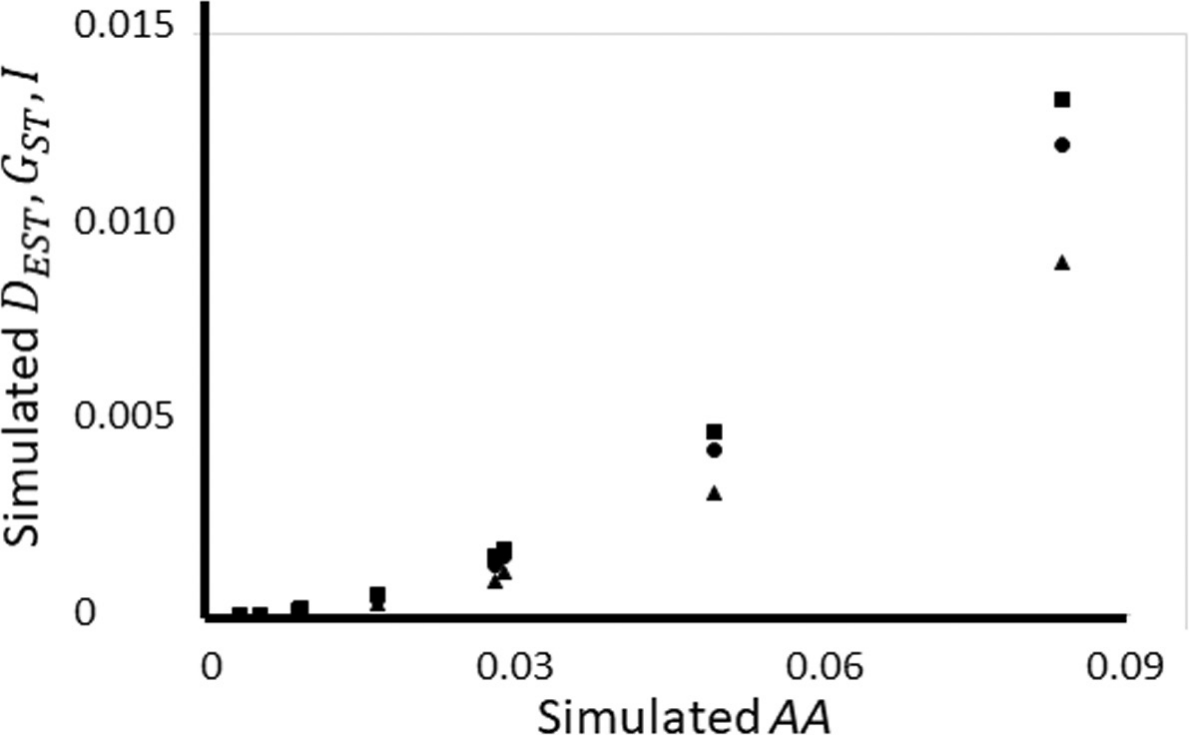
$D_{\mathrm{EST}},G_{\mathrm{ST}},\text{and}\;I$ (mutual information) plotted against ^*A*^ *A* (i.e., *BCAFD* corrected for maximum‐value dependency). $D_{\mathrm{EST}}$ is shown as squares, $G_{\mathrm{ST}}$ as discs, I as triangles. All measures were from the same simulated dataset that was used in Figure 1.

There are nonlinear relationships between ^*A*^ *A* and three other differentiation measures: $G_{\mathrm{ST}}$, $D_{\mathrm{EST}}$, and mutual information, *I*, as was suggested by a previous investigation of *BCAFD* (Berner, 2019a, 2019b) (Figure 3). This shows that ^*A*^ *A* provides information that is not linearly dependent on these other measures, which is important when using multiple measures for confirmation of results such as assessment of connectivity, and searches for loci potentially under selection.

As well as the equilibrium forecasts just described, it is important to have nonequilibrium forecasts for ^*A*^ *A*, which will often be relevant in many situations, including recently disturbed populations; nonequilibrium forecasts are shown in Equation (A1.11b).

It was also investigated whether the dependence of *BCAFD* on within location (alpha) variation could be fixed by basing the expectations for *BCAFD* not on $G_{\mathrm{ST}}$, but upon $G{"}_{\mathrm{ST}}$ (Meirmans & Hedrick, 2010). Unlike $G_{\mathrm{ST}}$, $G{"}_{\mathrm{ST}}$ is free of influence of within‐population variation. In Equation (A1.14), it can be seen that this new formulation of *BCAFD* is still heavily dependent upon heterozygosity *H*, including the within population (alpha) measures $H_{1}$ and $H_{2}$.

With the false detection of selection held constant at 1%, the important matter is the performance value: what percentage of loci that are classified as outliers, due to their differentiation value surpassing the univariate or bivariate criterion, are actually under selection—the true positives (TP). For a wide range of selection strengths, Table 2 shows the performance values for each univariate criterion (a single differentiation measure), and each bivariate criterion (i.e., an outlier locus must surpass the cutoff value for two differentiation measures). Of course, with the strongest selection (*s* = 0.05), all criteria performed well, but with very weak selection (*s* = 0.001), there was poor performance. The right‐hand column of Table 2 shows the performance averaged over all selection strengths, which had similar rankings for the performance of the criteria. The univariate criteria did not perform as well as the bivariates, with no overlap of mean performance ±1 × SE. Within the univariates, there was similar performance for all criteria, but when averaged over all selection strengths, the three best performers were ^*A*^ *A*, I, and $G_{\mathrm{ST}}$. Within the bivariate criteria again there was similar performance for all criteria. Nevertheless consistently the three best performers were “$D_{\mathrm{EST}}$ & I,” tied with “$D_{\mathrm{EST}}$ & $G_{\mathrm{ST}}$,” followed by “^*A*^ *A* & $G_{\mathrm{ST}}$.”

**TABLE 2:** Detection of loci under directional selection

Criteria {Differentiation measure(s)}	Known selection strength (*s*)				
	0.001	0.003	0.005	0.05	Mean performance
^***A***^ ***A***	488.18 ± 5.90 10 ± 0 33.03	851.18 ± 4.20 10 ± 0 46.23	911 ± 2.57 10 ± 0 47.92	999 ± 0.30 10 ± 0 50.23	**44.35 ± 3.86**
BCAFD	485.36 ± 6.15 10 ± 0 32.9	820.45 ± 4.69 10 ± 0 45.32	891 ± 3.16 10 ± 0 47.37	998.73 ± 0.38 10 ± 0 50.22	43.95 ± 3.82
$G_{\mathrm{ST}}$	459.91 ± 5.46 10 ± 0 31.72	857.55 ± 3.75 10 ± 0 46.42	938.09 ± 2.27 10 ± 0 48.65	999.82 ± 0.12 10 ± 0 50.25	**44.26 ± 4.25**
$D_{\mathrm{EST}}$	488.36 ± 6.57 10 ± 0 33.03	804.82 ± 4.54 10 ± 0 44.84	874.73 ± 3.73 10 ± 0 46.91	998.18 ± 0.40 10 ± 0 50.21	43.75 ± 3.74
I	458.27 ± 5.35 10 ± 0 31.64	857 ± 3.71 10 ± 0 46.4	938 ± 2.27 10 ± 0 48.65	999.82 ± 0.12 10 ± 0 50.25	**44.24 ± 4.27**
^*A*^ *A*, BCAFD	468.91 ± 5.52 7.45 ± 0.25 38.87	820.45 ± 4.69 7.45 ± 0.25 52.66	891 ± 3.16 7.45 ± 0.25 54.71	998.73 ± 0.38 7.45 ± 0.25 57.52	50.94 ± 4.15
^***A***^ ***A*,** $G_{\mathrm{ST}}$	443.64 ± 5.09 5.64 ± 0.24 44.28	843.82 ± 4.32 5.64 ± 0.24 60.18	910.73 ± 2.57 5.64 ± 0.24 61.99	999 ± 0.30 5.64 ± 0.24 64.15	**57.65 ± 4.53**
^*A*^ *A*, $D_{\mathrm{EST}}$	470.18 ± 5.87 7.18 ± 0.26 39.81	804.82 ± 4.54 7.18 ± 0.26 53.1	874.3 ± 3.73 7.18 ± 0.26 55.16	998.18 ± 0.40 7.18 ± 0.26 58.41	51.62 ± 4.08
^*A*^ *A*, I	442.18 ± 5.01 5.64 ± 0.24 44.19	843.36 ± 4.26 5.64 ± 0.24 60.17	910.73 ± 2.57 5.64 ± 0.24 61.99	999 ± 0.30 5.64 ± 0.24 64.15	57.63 ± 4.55
BCAFD, $G_{\mathrm{ST}}$	450.45 ± 5.67 5.64 ± 0.34 44.65	819.67 ± 4.77 5.64 ± 0.34 59.48	891 ± 3.16 5.64 ± 0.34 61.48	998.73 ± 0.38 5.64 ± 0.34 64.14	57.44 ± 4.37
BCAFD, $D_{\mathrm{EST}}$	475.55 ± 6.22 8.91 ± 0.16 35.03	804.82 ± 4.54 8.91 ± 0.16 47.71	874.73 ± 3.73 8.91 ± 0.16 49.79	998.18 ± 0.40 8.91 ± 0.16 53.09	46.40 ± 3.95
BCAFD, I	449 ± 5.56 6.18 ± 0.44 42.33	819.55 ± 4.71 6.18 ± 0.44 57.26	891 ± 3.16 6.18 ± 0.44 59.29	998.73 ± 0.38 6.18 ± 0.44 62.01	55.22 ± 4.41
$G_{\mathrm{ST}}$, $D_{\mathrm{EST}}$	441.36 ± 5.67 4.91 ± 0.31 47.59	804.09 ± 4.62 4.91 ± 0.31 62.32	874.73 ± 3.73 4.91 ± 0.31 64.28	998.18 ± 0.40 4.91 ± 0.31 67.25	**60.36 ± 4.38**
$G_{\mathrm{ST}}$, I	458.27 ± 5.35 10 ± 0 31.64	857 ± 3.71 10 ± 0 46.4	938 ± 2.26 10 ± 0 48.65	999.82 ± 0.12 10 ± 0 50.25	44.24 ± 4.27
$D_{\mathrm{EST}}$, I	439.91 ± 5.59 4.91 ± 0.31 47.51	804.09 ± 4.62 4.91 ± 0.31 62.32	874.73 ± 3.73 4.91 ± 0.31 64.28	998.18 ± 0.40 4.91 ± 0.31 67.25	**60.34 ± 4.40**

*Note:* The table shows the number of loci (±SE) from selection simulations of 1000 loci, which were identified as being under selection by criteria based on differentiation values from neutral simulations of 1000 loci: either a “univariate” criterion of being in the top 1% of neutral values for one differentiation measure, or a “bivariate” criterion of being simultaneously in the top 1% for two differentiation measures. In each of columns 2–5, the top value in each cell is the number of loci identified as being under selection (true positive, TP), in the selection simulation with the known value of selection shown at the top of the column, out of the total of 1000 independent loci simulated. The second value in each cell is the number of loci identified as being under selection (False positive, FP), in the parallel neutral simulation; of course with univariate criteria and the cutoff being the top 1%, the FP value is always 10 (1% of 1000 loci). The third value in each cell is the “performance” value—the percentage of loci that are true positive, out of all loci identified as outliers by that criterion (TP & FP). The performance value shown is for the case where 1% of all loci were under that selective regime, and all other loci were neutral; the calculation is 100 × (TP × 0.01)/[(TP × 0.01) + (FP × 0.99)]. Of course, the proportions of neutral and selected loci would not be known beforehand in a study designed to detect loci under selection, but given that it is standardized to a constant univariate FD rate, the performance values can be used to compare the criteria. The right column shows the performance averaged over all four selection strengths. Within each of the univariate criteria and the bivariate criteria, the three criteria with the best average performance are bolded. Note that the rank order of performance values is similar for most selection strengths, except the weakest selection (*s* = 0.001).

## 4 DISCUSSION

Science progresses by making forecasts under given conditions, then testing to see whether these conditions are confirmed by the data. Examples include assessing levels of dispersal by identifying whether neutral loci depart from expectations for isolation or panmixia, and testing for loci that may be responding to geographically variable selection, by identifying whether genetic differentiation is higher than neutral expectation (“outlier loci,” (Bierne et al., 2013; Lotterhos & Whitlock, 2014; Narum & Hess, 2011; Schneider et al., 2021; Xiang‐Yu et al., 2016)). Unfortunately, there are expected to be many false results in such molecular ecological methods (Bierne et al., 2013; Lotterhos & Whitlock, 2014; Narum & Hess, 2011; Schneider et al., 2021; Whitlock & McCauley, 1999; Xiang‐Yu et al., 2016). Therefore, it is advisable to confirm conclusions by methods that are mathematically independent or at least partially independent. Figure 3 shows that ^*A*^ *A* = $\frac{\left|p_{1}-p_{2}\right|}{\left(0.6152+0.3985\times p_{\max}\right)}$ (Equation 7) provides information that is not linearly dependent on three other differentiation measures: $G_{\mathrm{ST}}$, $D_{\mathrm{EST}}$, *I*, as previously suggested by an investigation of *BCAFD* (Berner, 2019a). ^*A*^ *A* is therefore complementary to $G_{\mathrm{ST}}$, $D_{\mathrm{EST}}$, *I*, and other measures (discussed below), and so it is a useful addition to our range of genetic differentiation measures, able to provide at least partly independent validation of results.

The forecasts in Equations (8) and (9) for ^*A*^ *A* can now be added to the armory of null expectations in assessment of connectivity and searches for loci under selection, because the forecasts for ^*A*^ *A* are very accurate in simulation results for the common data type of neutral biallelic SNPs, over a wide range of dispersal rates and effective population sizes (Figure 1b,c). If researchers do wish to use *BCAFD* and still achieve this accuracy, the researchers need to filter so that they use only those loci with maximum allele proportion (in either of the two locations) in the range 0.8 to unity, thus losing much of their dataset.

It is worth noting that ^*A*^ *A* (and *BCAFD*) are still dependent upon other aspects of within‐locality alpha‐variation, because of their relationship to $G_{\mathrm{ST}}$ and $F_{\mathrm{ST}}$. It was not possible to remove this dependence by basing the expectations for ^*A*^ *A* upon $G{"}_{\mathrm{ST}}$ (Meirmans & Hedrick, 2010) (Equation A1.14); moreover, such a correction would considerably complicate the derivation of theoretical expectations for ^*A*^ *A* or *BCAFD*, such as Equations (8) and (9). However, the alpha‐dependence is not fatal; despite their alpha‐dependence, $G_{\mathrm{ST}}$ and $F_{\mathrm{ST}}$ are frequently used in various ways, including assessing connectivity and searching for loci under geographically variable selection. Moreover, under some conditions $G_{\mathrm{ST}}$ and $F_{\mathrm{ST}}$ have performance comparable or better than other measures (Schneider et al., 2021). Nevertheless, like all such methods, there are expected to be many false‐positives, so that corroboration with semi‐independent assessments is needed (Bierne et al., 2013; Lotterhos & Whitlock, 2014; Narum & Hess, 2011; Schneider et al., 2021; Xiang‐Yu et al., 2016), which is where ^*A*^ *A* might be used.

The neutral forecasts for ^*A*^ *A* can be used either to make biological‐inventories of differentiation between locations (or times), or to be compared to observations in order to assess biological processes that underlie all biology, and are the processes which some conservation initiatives aim to conserve (Anonymous, 1988). Processes to be investigated include population size, mutation, and dispersal in natural or managed systems, or searches for outlier loci that depart from neutral expectations, and are thus candidate adaptive loci, which of course are very important in evolution and conservation (Teixeira & Huber, 2021). Candidate adaptive loci are identified because they depart from neutral forecasts, as is commonly done with $G_{\mathrm{ST}}$, $F_{\mathrm{ST}}$, and other measures (Bierne et al., 2013; Lotterhos & Whitlock, 2014; Narum & Hess, 2011; Schneider et al., 2021; Xiang‐Yu et al., 2016). Similar searches for adaptive loci are now using *BCAFD* (Bharti et al., 2021; Boyle et al., 2021; Haenel, Guerard, et al., 2021; Haenel, Oke, et al., 2021; Price et al., 2020; Yin et al., 2021; Zhou et al., 2021). These searches are expected to benefit from using ^*A*^ *A* instead of *BCAFD*, because as shown in the results above, the $p_{\max}$ limitation of *BCAFD* truncates the high values of differentiation, which are the very values used to identify the potentially adaptive loci.

Table 2 shows the performance of various criteria for identifying candidate adaptive loci under selection, due to their being outliers whose geographic genetic differentiation is in the top 1% of values for neutral loci for either a single measure (univariate criterion), or two measures (bivariate criterion). Of course, the more restrictive bivariate criterion eliminated more neutral loci, so the bivariate measures showed the best performance, measured as the percent of all outlier loci that were truly under selection (right column in Table 2). Table 2 indicates that there is no perfect measure for detecting selection, because when we are searching for loci under selection, we cannot know in advance the proportion of loci that are experiencing each selection strength. Nevertheless, it is reassuring to see that the rank order of the average performance over all selection strengths, is similar to the rank order within each selection strength. Of the univariate criteria, the three best performers were ^*A*^ *A*, I, and $G_{\mathrm{ST}}.$ The bivariate criteria generally performed much better, showing the advantage of using more that one differentiation measure as the cutoff in searches for candidate adaptive loci. Of the bivariate criteria, the three best performers were “$D_{\mathrm{EST}}$ & I,” tied with “$D_{\mathrm{EST}}$ & $G_{\mathrm{ST}}$,” followed by “^*A*^ *A* & $G_{\mathrm{ST}}$.” The differences in performance were small, but even small improvements are very important given that this commonly used approach can only identify outlier loci that are putatively under selection, then each of these “candidate” loci must be confirmed by separate extensive investigations, such as “evolve and resequence” experiments in one or more standard environmental conditions (Schlötterer et al., 2015).

There could be further research into which complementary measures are best to use with ^*A*^ *A*. This will depend upon the aim of the investigation and the different sensitivities of each measure, but some generalizations are possible. There has been considerable investigation of the properties of the Hill diversity measures, with many having good predictions from underlying factors such as population size, speciation/mutation, and dispersal, as well as showing independence of alpha, beta, and gamma (total) diversity (Sherwin et al., 2017, 2021). In particular, Shannon Mutual Information *I*/Shannon Differentiation and Morisita‐Horn/$D_{\mathrm{EST}}$ are differentiation measures that have available forecasts under neutrality that can be used as null models. These measures also avoid the dependency on within‐location (alpha) variation seen with $G_{\mathrm{ST}}$, and $F_{\mathrm{ST}}$; moreover, the Shannon measures avoid the heavy emphasis of effects of common variants, such as is seen with Morisita‐Horn and $D_{\mathrm{EST}}$ (Jost, 2008; Magurran, 2004; Sherwin et al., 2017; Sherwin et al., 2021). If the primary purpose of assessing differentiation is for identification of loci under selection, another good measure to contrast with identifications by ${\;}^{A}A$ would be $B^{\mathrm{GD}}$, which can be used at any level of the Hill‐family “Q,” and has a good sensitivity to selection, and is particularly appropriate for multi‐SNP haplotypes, which are not considered in the current work (Schneider et al., 2021).

Of course, any use of theory relies upon adherence to assumptions, and this initial theory for ^*A*^ *A* has assumptions like any theory. The equations for $G_{\mathrm{ST}}$, upon which the ^*A*^ *A* forecasts are based, rely on a number of assumptions (Neigel, 2002; Ochoa & Storey, 2021; Semenov et al., 2019; Whitlock & McCauley, 1999) and each of these needs to be investigated if it is proposed to apply Equations (8) or (9) to any particular case. First, it was assumed that there are only two locations, of approximately equal effective size, which may be the case especially in some conservation applications, but other possibilities would require further theory. Second, it was assumed that there is symmetric dispersal *m*, the same for both locations, so that addressing a source‐sink situation would require further theory based on the continent–island model. Third, it should be noted that unlike the Hill‐family of diversity measures, ^*A*^ *A* (or *BCAFD*) cannot currently be corrected for absence or under‐representation of rare alleles, due to incomplete sampling of individuals, by the Good‐Turing correction (Chao & Jost, 2015) (A. Chao pers. comm.); however, this correction method is also inapplicable to any measure based on a two‐variant system such as SNPs. Finally, Figure 1b shows a wide scatter, but the regression analysis shows that if there are multiple independent replicates such as hundreds, or a thousand, neutral SNP loci in linkage equilibrium, the neutral forecast is very accurate. This number of statistically unlinked SNP loci is easily achievable with current methods for genotyping‐by‐sequencing (e.g., www.diversityarrays.com).

Irrespective of whether one wishes to use theoretical expectations, it is advisable to use ^*A*^ *A* rather than *BCAFD*, because the latter's dependence on $p_{\max}$ limits its comparability to other studies, even within the same species, if the population pairs analyzed are in parts of the range that have different $p_{\max}$, due to a strong cline.

Several further possible developments are obvious. First, Appendix 1 principally shows equilibrium forecasts; Tables A2.1 and 2.2 show that there is often a wide range of generation times for which equilibrium is a reasonable assumption. However, there are populations that are known to have had recent changes such as severe reductions in connectivity, and for these the Equation (A1.11b) can be used. For other changes such as reduction of population size, further nonequilibrium forecasts could be derived in later research. Second, the initial neutral theory of ^*A*^ *A* in this article gives a good null model for use in searches for outlier loci that may be under directional selection, but could form the basis of further theory that is specific to particular modes of selection, including more complicated geographical patterns of directional selection, or balancing or disruptive selection. Third, at present the theory is limited to cases where there are only two alleles, as is often the case for SNPs, but not for haplotypes encompassing many nucleotides. In future, all the theory in this paper might be extended to cases with multiple alleles, broadening it to encompass the multiallele version of *BCAFD* ((Berner, 2019a) Supplement). Fourth, the theory could be extended to multiple locations. Fifth, the haploid Equation (8) might also be developed to deal with species variants in two local communities, if the speciation rate is negligible relative to the dispersal rate; this is of course the original use of Bray‐Curtis (Bray & Curtis, 1957), which would require development of multivariant theory plus simulations tailored to species assemblages, including investigation of the wide scatter seen in Figure 1, for which species analyses could not be overcome by using hundreds or more replicate loci—instead, hundreds or more replicate pairs of communities would be needed, which is probably unattainable.

In conclusion:

- The new ^*A*^ *A* measure (Equation 7) provides a semi‐independent means for assessing connectivity, selection, etc. based on geographic genetic differentiation, that can be used in combination with other such measures to minimize errors such as false positives.
- The ^*A*^ *A* measure avoids counterintuitive truncation of high values of beta‐differentiation by alpha within‐population variation ($p_{\max}$),
- Avoiding this truncation means that that studies with different $p_{\max}$ can now be compared realistically, either between species, or even within the same species, if the population pairs analyzed are in parts of the range that have different $p_{\max}$, due to a strong cline.
- Avoiding this truncation is especially important if the high values of differentiation are to be used to identify candidate adaptive loci, because the truncation would pull the truly high values in amongst the not‐quite‐so‐high, leading to increased false negatives and positives.
- As predicted, the best performance at identifying outlier loci that are potentially under selection comes from using two geographic genetic differentiation measures simultaneously, to make bivariate criteria; the three best performers were “$D_{\mathrm{EST}}$ & I,” tied with “$D_{\mathrm{EST}}$ & $G_{\mathrm{ST}}$,” followed by “^*A*^ *A* & $G_{\mathrm{ST}}$.” The differences in performance are very important given that each of the identified “candidate” loci must be confirmed by separate extensive investigations
- As well as simply presenting patterns in the data, if researchers consider that their system conforms to the assumptions herein, the neutral forecasts for ^*A*^ *A* can be used as a rigorous basis for investigations such as tests for selection and assessment of connectivity.
- There are equilibrium and nonequilibrium versions of the theory for ^*A*^ *A* (Equations 8, 9, A1.11b).
- Irrespective of whether the theory in this paper is used, *BCAFD* cannot be free of the limit of maximum within‐population allele proportion $p_{\max}$, so it is best if reported differentiation values should be based upon ^*A*^ *A*, not BCAFD.

## AUTHOR CONTRIBUTIONS

**William B. Sherwin:** Conceptualization (lead); data curation (lead); formal analysis (lead); investigation (lead); methodology (lead); project administration (lead); resources (lead); software (lead); validation (equal); visualization (lead); writing – original draft (lead); writing – review and editing (lead).

## CONFLICT OF INTEREST

There are no conflicts of interest.

## Figures and Tables

**FIGURE A2.1:**
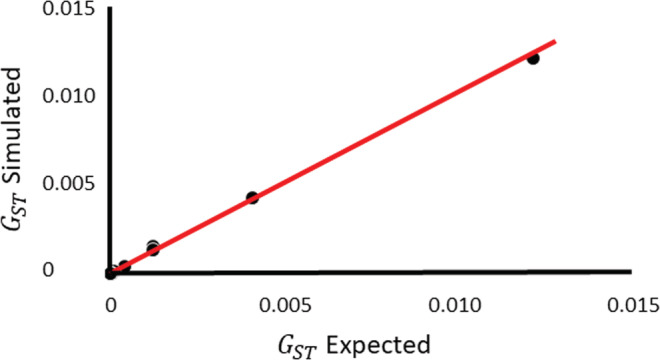
Comparison of simulation results with algebraic predictions for $G_{\mathrm{ST}}$, with regression equation (Simulated‐$G_{\mathrm{ST}}$) = 0.97 × (Predicted‐$G_{\mathrm{ST}}$) + 0.00005; *P* = 4.2 × 10^−12^; *R* ^2^ = .99. Predicted $G_{\mathrm{ST}}$ is from Equation (A1.5b) (Takahata, 1983), using 4 *N* in place of 8 *N* for the haploid loci simulated. The intercept is very close to zero as expected. The slope coefficient (0.97) has 95% confidence limits 0.95 to 1.00. The red line is the expected 1:1 relationship, indistinguishable from the actual regression line. Note that for each scenario, the coefficient of variation for simulated $G_{\mathrm{ST}}$ was always larger than the coefficient of variation for ^*A*^ *A* (sometimes double).

## Data Availability

MATLAB program, and data, are on DRYAD at https://doi.org/10.5061/dryad.2547d7wt7

## APPENDIX 1 Forecasting equilibrium Bray‐Curtis with mutation, dispersal, and drift due to small population size, for two locations, with a single neutral biallelic SNP locus

There are two locations with indices *i* = 1,2. Where there is no index, or the index is T, it is the value calculated for the pooled locations (metapopulation), for example, pooled allele proportion, overall heterozygosity.

^*A*^ *A—*“Adjusted‐AFD,” i.e., Bray‐Curtis between locations “1” and “2”, adjusted to compensate for the limitation that *BCAFD* cannot be greater than $p_{\max}$ the maximum allele proportion in either of the two locations: ^*A*^ *A* = $\frac{\left|p_{1}-p_{2}\right|}{\left(0.6152+0.3985\times p_{\max}\right)}$

*BCAFD—*Bray‐Curtis between locations “1” and “2”, the unsigned difference of proportions, that is, $\text{BCAFD}=\left|p_{1}-p_{2}\right|$ (Berner, 2019a, 2019b) (Equation 2 in main article). (This is also called *AFD*—Difference of Allele “Frequency” i.e., proportion). The algebra below deals with a single locus, but *BCAFD* can be averaged over loci.

${\;}^{2}D$—Second order Hill diversity, or effective number of alleles ${\;}^{2}D=1/\left(1-H\right)$ or $H=1-1/{\;}^{2}D=\frac{{\;}^{2}D-1}{{\;}^{2}D}$

*F*_*ST*_—Wright's measure of differentiation for biallelic SNPs

$G_{\mathrm{ST}}=F_{\mathrm{ST}}=\sigma_{p}^{2}/\bar{p}\;\bar{q}=\left[H_{T}-\bar{H_{1},H_{2}}\right]/H_{T}$ ([Halliburton, 2004] Box 9.5)

*G*_*ST*_—See *F*_*ST*_; these are equivalent in the 2‐allele, 2‐location case.

H—Binomial (Hardy–Weinberg) expected heterozygosity, for example, $H_{T}=1-{\bar{p}}^{2}-{\bar{q}}^{2}$; $H_{1}=1-p_{1}^{2}-q_{1}^{2}$

*i* = 1,2,—indices for the two members of a pair of locations. Where there is no index, or the index is T, it is the value calculated for the pooled locations (metapopulation), for example, pooled allele proportion, overall heterozygosity.

*k* —number of localities (always two unless stated otherwise)

*m*—dispersal per generation between the two populations, symmetrical (0 ≤ *m* ≤ 1)

*N*—effective population size at each location (identical)

*p*_*1*_ *p*_*2*_—proportions of the chosen allele at each location “*i*” ($0\leq p_{i}\leq 1$) at generation *t*; for the other allele, *q*_*i*_ = 1 − *p*_*i*_.

$\bar{p}$—average *p* over the two locations at beginning of generation *t*: $\bar{p}=\left(p_{1}+p_{2}\right)/2$; $\bar{q}=1-\bar{p}$

*p′*—proportions partway through generation *t*.

*p″*—proportions one generation after time *t* (at time *t″*).

*t*—generation index (*t″* after one full generation).

T—is the index for the pooled locations (metapopulation), for example, overall heterozygosity.

μ—mutation rate per generation (0≤μ≤1)

$\sigma_{p}^{2}$—Variance of *p* between locations 1 and 2: $\sigma_{p}^{2}=\bar{p_{i}^{2}}-{\left(\bar{p_{i}}\right)}^{2}$

I restricted analysis to cases where there are two locations:

- with identical effective population size,
- reproduction with stochastic drift in each population is followed by dispersal
- deterministic symmetric dispersal between the two locations
- two alleles per locus (e.g., conventionally filtered SNP data)

These are shown schematically in Table A1.1, for a single generation.

**TABLE A1.1:** Scheme for the simulation, for each generation, using terms defined in text of Appendix 1

	Location 1	Location 2
Generation *t*, initially	*p*_1_, *q*_1_	*p*_2_, *q*_2_
After drift	$p_{1}^{\prime },q_{1}^{\prime }$	$p_{2}^{\prime },q_{2}^{\prime }$
After dispersal	$\begin{array}{l} p_{1}^{\prime \prime }=p_{1}^{\prime }-mp_{1}^{\prime }+m^{}p_{2}^{\prime }\\ q_{1}^{\prime \prime }=1-p_{1}^{\prime \prime } \end{array}$	$\begin{array}{l} p_{2}^{\prime \prime }=p_{2}^{\prime }-mp_{2}^{\prime }+mp_{1}^{\prime }\\ q_{2}^{\prime \prime }=1-p_{2}^{\prime \prime } \end{array}$

### 1 BRAY‐CURTIS/AFD “*BCAFD*” AT DRIFT‐DISPERSAL EQUILIBRIUM

*BCAFD* between locations “1” and “2,” is (Berner, 2019a, 2019b)

(2 and A1.1)
$$\text{BCAFD}=|p_{1}-p_{2}|$$

At any time, for 2 localities with 2 alleles per locus,

(A1.2)
$$G_{\mathrm{ST}}=F_{\mathrm{ST}}=\left[H_{T}-\bar{H_{1},H_{2}}\right]/H_{T}=\sigma_{p}^{2}/\bar{p}\;\bar{q}$$

where $\sigma_{p}^{2}=\bar{p_{i}^{2}}-{\left(\bar{p_{i}}\right)}^{2}$ ((Halliburton, 2004) Box 9.5 (Falconer & Mackay, 1996) equation 3.4)

(A1.3)
$$\mathrm{IE}\;\sigma_{p}^{2}=\left[\left(p_{1}^{2}+p_{2}^{2}\right)/2\right]-\left[{\left(\frac{\left(p_{1}+p_{2}\right)}{2}\right)}^{2}\right]={\left(p_{1}-p_{2}\right)}^{2}/4={\text{BCAFD}}^{2}/4$$

So $G_{\mathrm{ST}}=F_{\mathrm{ST}}={\text{BCAFD}}^{2}/4\bar{p}\;\bar{q}$

Or

(A1.4)
$${\text{BCAFD}}^{2}=4\bar{p}\;\bar{q}G_{\mathrm{ST}}=2H_{T}G_{\mathrm{ST}}$$

Now with diploid individuals at dispersal‐drift‐mutation equilibrium for *k* localities, it is expected that (equations 8 and 20 in [Takahata, 1983])

(A1.5a)
$$G_{\mathrm{ST}}=1/\left(1+\frac{4k}{k-1}\left(\mathrm{N\mu}+\frac{\mathrm{kNm}}{k-1}\right)\right)$$

so with one pair of localities, k=2, then

(A1.5b)
$$G_{\mathrm{ST}}=1/\left(1+8N\left(2m+\mu \right)\right)$$

So inserting Equation (A1.5b) into Equation (A1.4), at equilibrium,

(A1.6)
$$\begin{array}{c} {\text{BCAFD}}^{2}=2H_{T}/\left(1+8N\left(2m+\mu \right)\right)=\frac{2{\;}^{2}D-2}{{\;}^{2}D\left(1+8N\left(2m+\mu \right)\right)} \end{array}$$

We get for diploid:

(A1.7)
$$\text{BCAFD}=\sqrt{\frac{2{\;}^{2}D-2}{{\;}^{2}D\left(1+8N\left(2m+\mu \right)\right)}}$$

And for haploid:

(A1.8)
$$\text{BCAFD}=\sqrt{\frac{2{\;}^{2}D-2}{{\;}^{2}D\left(1+4N\left(2m+\mu \right)\right)}}$$

### 2 DYNAMIC (NONEQUILIBRIUM) ^*A*^ *A* OVER TIME AFTER DISPERSAL IS REDUCED TO ZERO

The equilibrium calculations presented above are appropriate in many cases, with Tables A2.1 and A2.2 below showing that there is usually a wide window of generation times for which equilibrium is a reasonable assumption. However, in both natural and modified habitats, often there is a nonequilibrium situation such as a sudden reduction in connectivity, for example, due to new human infrastructure. Therefore, dynamic (nonequilibrium) equations are also needed, and one such equation is derived below to give Equation (A1.11b), for time t generations after a complete cessation of dispersal between two locations.

At time *t* after a diploid population is split into two subpopulations with zero dispersal between them ((Falconer & Mackay, 1996) equation 3.2):

(A1.9)
$$\sigma_{p}^{2}\left(\mathrm{at}\;\text{time}\;t\right)=\bar{p_{\text{init}}}\;\bar{q_{\text{init}}}\left[1-{\left(1-1/2N\right)}^{t}\right]$$

where $\bar{p_{\text{init}}}$ and $\bar{q_{\text{init}}}$ are the average allele proportions immediately before the split

From Equation (A1.3) above,

(A1.10)
$$\sigma_{p}^{2}={\;}^{A}A^{2}/4\;\text{or}\;{\;}^{A}A=\sqrt{4\;\sigma_{p}^{2}}$$

Note the use of ^*A*^ *A* rather than *BCAFD* in these dynamic equations, because they are modeling complete isolation, which will result in approach to high differentiation, which the main article shows is best forecast for ^*A*^ *A*, not *BCAFD*. If we are averaging over many loci, it is reasonable to assume that average allele proportions for the metapopulation ($\bar{p_{\text{init}}}$ and $\bar{q_{\text{init}}}$) do not change over time. Then at time *t* after dispersal is reduced to zero, combine Equations (A1.9) and (A1.10):

(A1.11a)
$${\;}^{A}A\;\left(\mathrm{at}\;\text{time}\;t\right)=\sqrt{4\bar{p_{\text{init}}}\;\bar{q_{\text{init}}}\;\left[1-{\left(1-1/2N\right)}^{t}\right]}$$

i.e.,:

(A1.11b)
$${\;}^{A}A\;\left(\mathrm{at}\;\text{time}\;t\right)=\sqrt{2H_{T\;\left(\text{init}\right)}\left[1-{\left(1-1/2N\right)}^{t}\right]}$$

In Equation (A1.11a), (A1.11b), for haploids, 2 *N* is replaced by *N*.

### 3 CAN WE CORRECT FOR DEPENDENCE ON ALPHA AND GAMMA?

Note that due to the dependence of $G_{\mathrm{ST}}$ on (alpha) heterozygosity, *BCAFD*, and derived measures such as ^*A*^ *A*, are also expected to be dependent upon (alpha) heterozygosity, and such dependence is not a desirable property for a measure of between‐locality (beta) differentiation. This is additional to the dependence of *BCAFD* on maximum allele proportion, for which a correction is applied in the main article (^*A*^ *A*). However, there is a correction for the unwanted dependency of $G_{\mathrm{ST}}$ on (alpha) heterozygosity (Meirmans & Hedrick, 2010), so it is interesting to ask whether using this correction would remove unwanted dependency for *BCAFD* and thus ^*A*^ *A*. For a pair of locations, the corrected $G_{\mathrm{ST}}$ is:

(A1.12)
$$G_{\mathrm{ST}}^{\prime \prime }=\frac{2\left(H_{T}-\bar{H_{1},H_{2}}\right)}{\left(2H_{T}-\bar{H_{1},H_{2}}\right)\left(1-\bar{H_{1},H_{2}}\right)}$$

Combining Equations (A1.2) and (A1.12),

(A1.13)
$$G_{\mathrm{ST}}=\frac{\left(2H_{T}-\bar{H_{1},H_{2}}\right)\left(1-\bar{H_{1},H_{2}}\right)}{2H_{T}}G_{\mathrm{ST}}^{\prime \prime }$$

Combining Equations (A1.4) and (A1.13)

(A1.14)
$${\;}^{A}A=\sqrt{\left(2H_{T}-\bar{H_{1},H_{2}}\right)\left(1-\bar{H_{1},H_{2}}\right)\;G_{\mathrm{ST}}^{\prime \prime }}$$

Although $G_{\mathrm{ST}}^{\prime \prime }$ is free of alpha‐dependency, nevertheless this new formulation of ^*A*^ *A* is clearly still heavily dependent upon heterozygosity ($H_{T},\bar{H_{1},}\bar{H_{2}}$), which are gamma and alpha measures, which would ideally not affect a (beta) differentiation measure. Additionally, using this formulation in Equation (A1.14) for *BCAFD* would considerably complicate the derivation of theoretical expectations comparable to Equations (A1.7) and (A1.8).

## APPENDIX 2 The MATLAB simulation program

This MATLAB program was modified from the one previously described (Dewar et al., 2011), to include calculation of the Bray‐Curtis/AFD Index *BCAFD* and ^*A*^ *A* (Equations 2, 7, A1.1), as well as the previously calculated $G_{\mathrm{ST}}$ (Equation A1.2), $D_{\mathrm{EST}},\text{and}\;I$ (mutual information).

The simulation deals with two biallelic haploid subpopulations, for scenarios with every possible combination of levels of three symmetric dispersal rates (*m* = 0.01, 0.03, 0.1) and three effective subpopulation sizes (*N* = 1000, 10, 000, 100, 000), giving a total of nine scenarios. Mutation rate between the two alleles per SNP locus per generation was held at *μ* = 10^−9^, a likely rate for SNP alleles, but preliminary simulations showed virtually identical results with the much higher and lower rates of *μ* = 10^−6^ and 10^−12^. Indeed, inspection of Equations (A1.7) and A1.8 show that mutation rate will be largely irrelevant unless the locations are completely isolated (*m* = 0 exactly). Starting allele proportions in each subpopulation ($p_{1},p_{2}$) were randomized for each subpopulation in each replicate of each scenario (0≤p≤1). Each generation included stochastic binomial sampling of the available parent alleles to establish the allele proportions for the offspring, followed by deterministic symmetrical dispersal to create the parent populations for the next generation. For each scenario (combination of *m*, *N*), there were 1000 independent replicate iterations, which could be regarded either as 1000 different pairs of populations, or one pair of populations with 1000 SNP loci showing independent segregation (i.e., in linkage equilibrium); such data are now commonplace. Note that researchers using SNP data typically search for linkage disequilibrium between pairs of loci, and remove one of each pair. If researchers obtain less than 1000 SNP loci that are unlinked (Waples et al., 2022), this will reduce the precision of any differentiation measure, including $G_{\mathrm{ST}}$ or ^*A*^ *A*. Note that if there is moderate to high recombination, the number of chromosomes does not directly limit the number of statistically unlinked loci. Each iteration was run for 200 generations. Because the calculations in Appendix 1 are for equilibrium given values of *m*, *μ*, and *N*, it was important to ensure that this number of generations was long enough to allow a close approach to equilibrium. This was ensured in three ways. First, results were inspected to ensure that each scenario had asymptoted to a stable value for *BCAFD*, well before the final generation. Second, iterations were each also inspected to ensure that the variance of *BCAFD* between‐generations was much lower than variance between replicate iterations (typically one tenth or less). Finally, 200 generations was much greater than the expected time for $F_{\mathrm{ST}}$ to reach half drift‐dispersal equilibrium ($t_{1/2\;\mathrm{eq}}$ generations), which for diploids is (Crow & Aoki, 1984; Whitlock, 1992):

(A2.1a)
t1/2eq=ln0.5ln1−m21−12N

and for haploids is

(A2.1b)
t1/2eq=ln0.5ln1−m21−1N

where symbols are as in Appendix 1. Maximum time to half‐equilibrium is 34 generations for the scenarios trialed in the main paper (Table A2.1). Given that *BCAFD* is a function of $F_{\mathrm{ST}}$ or $G_{\mathrm{ST}}$, is seems reasonable to assume that this will also approximate the time to half‐equilibrium for *BCAFD* and ${\;}^{A}A$. The simulations should be run for several times this $t_{1/2\;\mathrm{eq}}$. For all simulated scenarios, a time of 200 generations was chosen, which is well in excess of the expected times to half‐equilibrium in Table A2.1.

**TABLE A2.1:** Time in generations to half‐equilibrium $t_{1/2\;\mathrm{eq}}$ for the scenario conditions simulated

*N*	*m*	$t_{1/2\;\mathrm{eq}}$
1000	0.01	32.8488
1000	0.03	11.1944
1000	0.10	3.27386
10,000	0.01	34.3131
10,000	0.03	11.3596
10,000	0.10	3.28785
100,000	0.01	34.4666
100,000	0.03	11.3764
100,000	0.10	3.28925

*Note:* See Appendix 1 for definitions of other symbols.

There is a second, opposing, constraint on the number of generations. As well as the need to ensure close approach to equilibrium, the calculations in Appendix 1 assume no fixation (i.e., loss of all alleles except one), so that it was important to run the simulations for times that are short enough to avoid fixation. This is also important because most researchers, or the companies that do their genotyping, will filter out invariant (fixed) SNPs from the data. Table A2.2 shows that it is possible to choose simulation generation numbers that are short enough to give minimum fixation, but sufficiently large to give approximate equilibrium (Table A2.1). In the case of two equal‐sized subpopulations making up a metapopulation with dispersal, *N* for metapopulation ≈2 × *N*‐subpopulation; for haploid we use 4 *N(metapop)* instead 8 *N* in the equation of expected time to fixation, and then we find that $t_{\mathrm{fix}}$ generations is given by ([Kimura & Ohta, 1969, Maruyama, 1970, Crow & Kimura, 1970] equation 8.9.4 p 431):

(A2.2)
$$t_{\mathrm{fix}}=-\frac{4\text{Npln}\left(p\right)}{1-p}$$

where symbols are as in Appendix 1. Times to fixation in generations, for the scenarios trialed in main paper, are shown in Table A2.2. This table shows that even for the smallest effective population size of 1000, fixation in about 200 generations would require a mean initial *p* of 0.01 or less, for example, initial $p_{1}=p_{2}=0.01$. With random assignment of initial *p* values in each of the two locations, such a situation would arise in only 0.01 × 0.01 × 100% = 0.01% of replicates. In an extreme case where *N* for the metapopulation was equal to the *N* for either subpopulation, the fixation times would be halved, and yet most of the fixation times would still be orders of magnitude larger than the 200 generations simulated.

**TABLE A2.2:** Expected time in generations to fixation for the scenario conditions simulated

Initial *p*	*N*	Fixation time
0.5	100,000	277258.9
0.1	100,000	102337.1
0.01	100,000	18606.7
0.5	10,000	27725.9
0.1	10,000	10233.7
0.01	10,000	1860.7
0.5	1000	2772.6
0.1	1000	1023.4
0.01	1000	186.1

*Note:* See Appendix 1 for definitions of symbols.

In case any fixation did occur, the program included a trap for fixation, and it was designed so that if fixation occurred in any iteration, then the iteration would be replaced by restarting from generation zero, in line with the filtering normally applied to such data. Because of the relatively short number of generations (200), there were virtually no restarts for fixation.

The simulations used a binomial mechanism for transmission of alleles between generations, because of the initial focus on 2‐allele SNPs. However other mechanisms such as Poisson or negative binomial might give different results (Warton & Hui, 2017), and this might be appropriate in other cases outside the scope of this paper, including where the underlying biological process for transmitting variants is different, or not adequately understood at present.

The accuracy of the simulations was tested in two ways:

- By limited comparison with results of EASYPOP (Balloux, 2001), in which the starting proportions could be set to be approximately $p_{1}=p_{2}=0.5$. These showed almost identical results for $G_{\mathrm{ST}}$ when the MATLAB simulation was run with initial $p_{1}=p_{2}=0.5\;\text{exactly}$.
- By checking $G_{\mathrm{ST}}$ results from the simulation against the well‐known forecasts from Equation (A1.5b). The results of the simulation showed very good fit to these forecasts over the wide range of conditions in the simulation. (Figure A2.1)
